# Supplementary material for: Peer Relationships and Psychosocial Difficulties in Adolescents: Evidence from a Clinical Pediatric Sample
Source: J Clin Med. 2025 Oct 11;14(20):7177. doi: 10.3390/jcm14207177 (PMC12565466; doi:10.3390/jcm14207177)
Supplement: Supplementary file 1 [file jcm-14-07177-s001.zip › jcm-3860182-supplementary.pdf]

Supplementary materials – S1 Additional details supporting the analyses reported in the main text

The following supplementary materials provide additional details supporting the analyses reported in the main text. Specifically, they include:

(1) the distribution of the SDQ Total Difficulties (SDQ TD) score;
(2) the correlation matrix of the study variables;
(3) diagnostic plots and formal diagnostic tests (including tests of normality, independence, and homoscedasticity, checks for multicollinearity, and influence diagnostics);
(4) complete regression outputs (OLS and robust MM) for SDQ TD and SDQ TD15; and
(5) model fit statistics (OLS and robust MM) for SDQ TD and SDQ TD15.

Figure S1. Distribution of SDQ Total Difficulties score (SDQ TD)

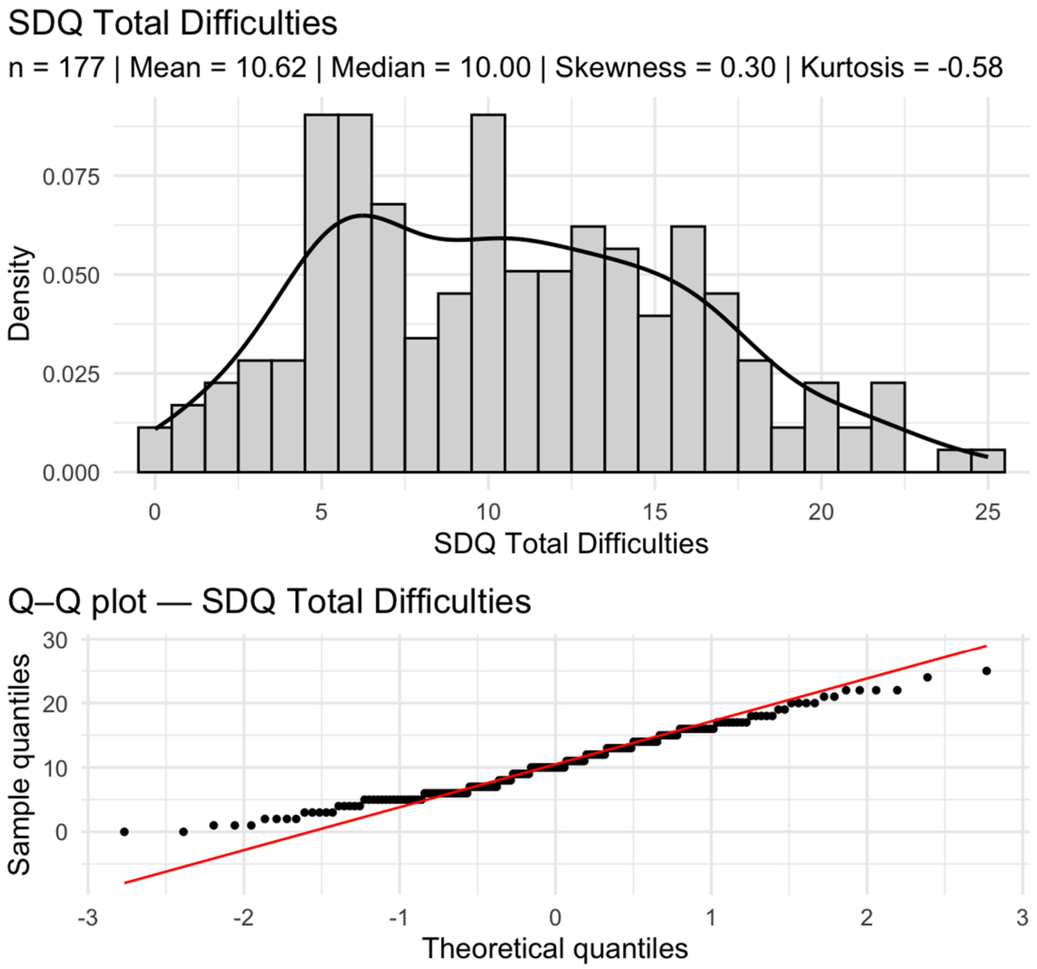

Note. Histogram and Q–Q plot of the SDQ TD (n = 177). Skewness and kurtosis were computed using the unbiased sample moment estimators. The theoretical score range is 0–40, but the observed range was 0–25. Although the Shapiro–Wilk test indicated a departure from normality (p = .006), deviations from the reference line were modest, supporting treatment of the outcome as approximately continuous.

Table S1. Correlation matrix among study variables (*p*-values BH-adjusted)

Variables	1	2	3	4	5	6	7	8	9	10	11	12	13	14	15	16	17	18
1. Sex	1																	
2. Age	−.13	1																
3. Chronic condition	−.08	−.05	1															
4. Italian citizen	−.08	−.02	.03	1														
5. Mother-relat. satisfaction	.00	**−.24**	−.02	−.03	1													
6. Father-relat. satisfaction	−.07	**−.31**	−.02	.03	**.69**	1												
7. Friendship satisfaction	−.09	.06	.10	.04	.15	.12	1											
8. Prosocial score	.11	−.08	−.05	.04	**.30**	**.32**	.12	1										
9. Eating disorder risk	.14	.01	−.04	−.07	.13	.01	−.04	−.02	1									
10. Alcohol lifetime	.05	.13	−.00	.13	−.12	**−.18**	−.02	**−.19**	**.23**	1								
11. Alcohol past year	.02	**.21**	−.04	.11	−.06	−.10	.06	**−.21**	**.25**	**.80**	1							
12. Alcohol past 30 days	.04	.17	−.11	.09	−.11	−.17	.04	**−.22**	**.34**	**.63**	**.80**	1						
13. Nicotine lifetime	−.14	**.35**	.00	.09	−.12	**−.21**	**.18**	−.16	.17	**.35**	**.41**	**.35**	1					
14. Nicotine past year	−.13	**.33**	−.00	.08	−.01	**−.20**	**.24**	−.15	**.24**	**.38**	**.43**	**.35**	.87	1				
15. Nicotine past 30 days	−.07	**.33**	−.02	.08	−.01	**−.25**	**.19**	−.11	**.20**	**.30**	**.33**	**.29**	**.84**	**.88**	1			
16. Cannabis lifetime	−.03	.06	−.11	.04	.04	−.09	.07	**−.19**	.03	.04	.08	.11	**.25**	**.29**	**.30**	1		
17. Cannabis past year	.01	.15	−.05	.03	.00	−.17	.11	−.18	.07	.08	.11	.15	**.32**	**.36**	**.38**	**.81**	1	
18. Cannabis past 30 days	.01	.15	−.05	.03	.00	−.17	.11	−.18	.07	.08	.11	.15	**.32**	**.36**	**.38**	**.81**	**1.00**	1

Note. Values are Spearman's ρ (continuous–continuous) or rank–biserial correlations (binary–continuous). *p*-values were adjusted within-matrix using the Benjamini–Hochberg procedure (FDR q = .05). The correlation between Cannabis past year (17) and Cannabis past 30 days (18) is exactly 1.00 because both variables identify the same subset of participants.

Figure S2. Component-plus-residual (partial) plots for continuous predictors in the OLS model with SDQ TD as the outcome

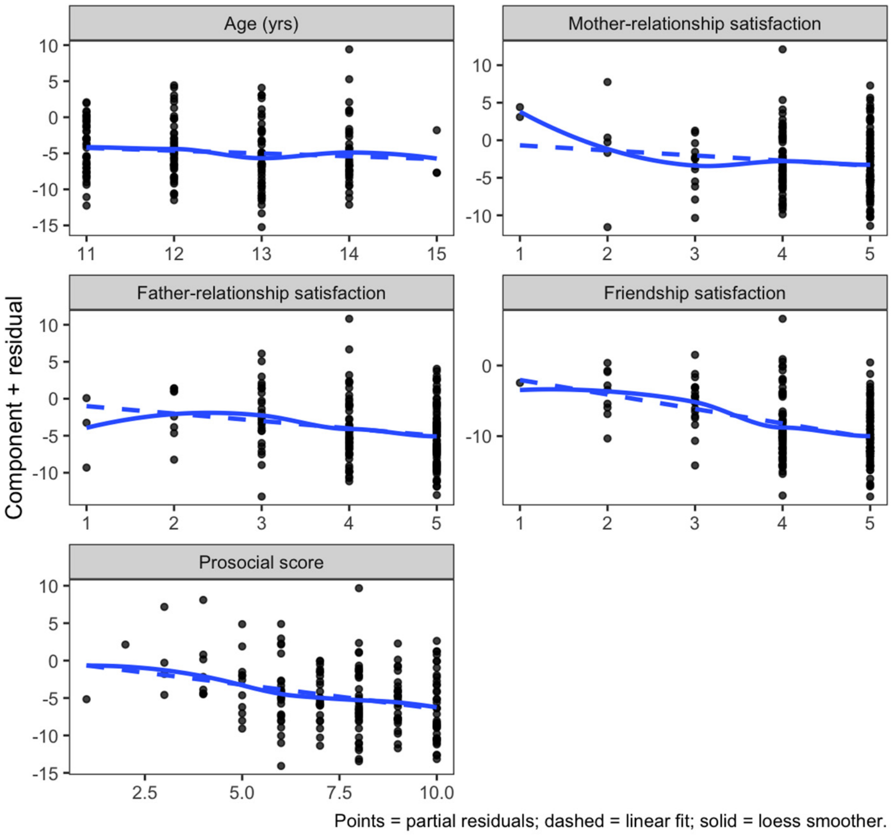

Note. Points are partial residuals; dashed line indicates the linear fit; solid line is loess smoother. No systematic curvature is evident, consistent with RESET $F(2,163) = 0.17$, $p = .840$).

Figure S3. Standard diagnostic plots for the OLS regression model with SDQ TD as the outcome: residuals *vs.* fitted values, scale–location plot, Q–Q plot of residuals, and residuals *vs.* leverage

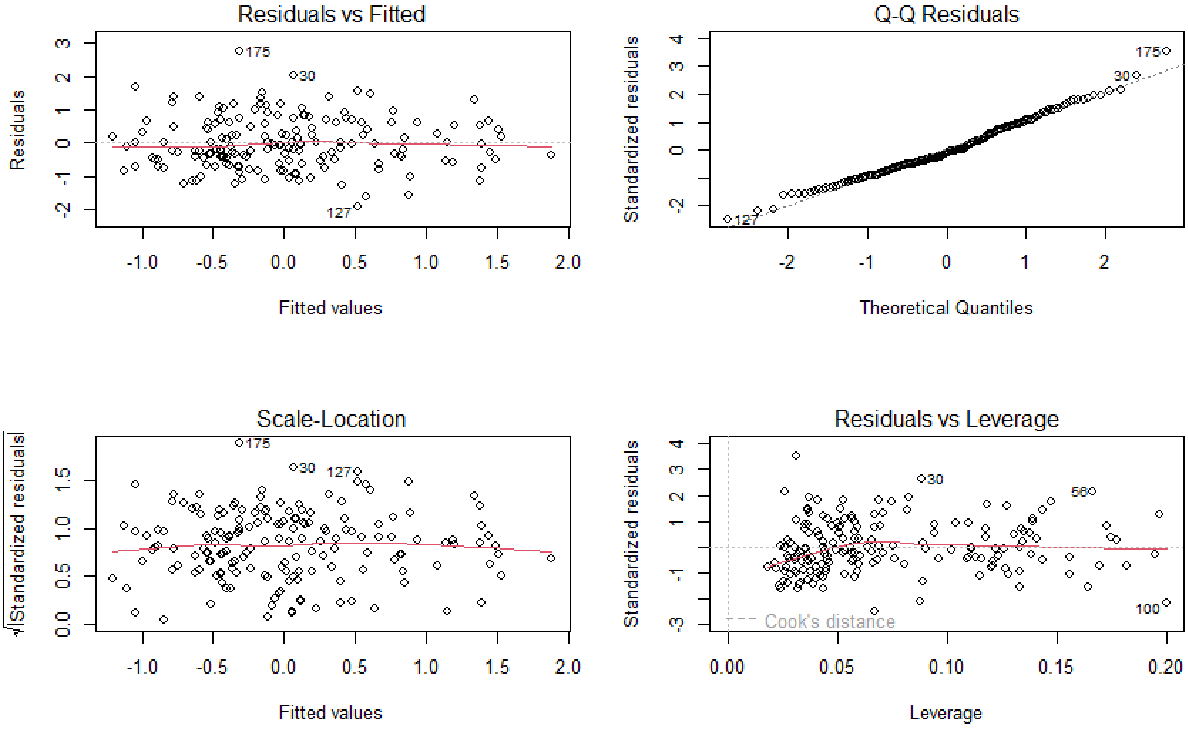

Figure S4. Influence diagnostics thresholds and counts for OLS model

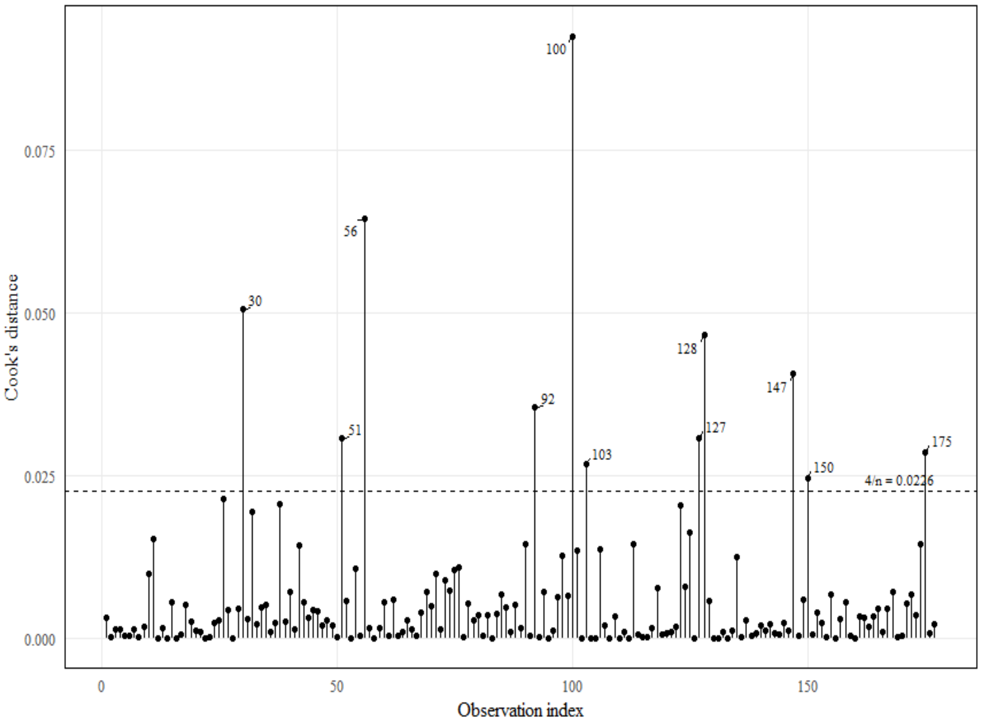

Table S2. OLS model diagnostic tests (SDQ TD)

Diagnostic test	Statistic	*p*-value
Shapiro–Wilk (SW)	0.987	.092
Durbin–Watson (DW)	1.941	.288
Breusch–Pagan (BP)	6.713	.822

Note. SW = Shapiro–Wilk normality test; DW = Durbin–Watson test for autocorrelation of residuals; BP = Breusch–Pagan test for heteroscedasticity

Table S3. OLS model influence diagnostics (SDQ TD)

Measure	Threshold	*n* cases exceeding
Cook's Distance	0.023	11
Leverage	0.136	16
DFFITS	0.520	7
DFBETAS	0.150	34
|Studentized residual|> 3	3	1

Note. Thresholds were calculated according to Stevens (2009). Cook's Distance = Overall influence of a case on all regression coefficients; combines residual size and leverage; Leverage = How far a case's predictors are from the center of the predictor space; DFFITS = Influence of a case on its own fitted value; combines residual and leverage for each case; DFBETAS = Influence of a case on each individual regression coefficient; |Studentized residual|> 3 = = Cases with unusually large standardized residuals (> 3), indicating potential outliers in the outcome variable given the model.

Reference: Stevens, J.P. (2009) *Applied Multivariate Statistics for the Social Sciences*. 5th Edition, Routledge, New York.

Table S4. Variance inflation factors (VIF) for predictors in the OLS model (SDQ TD)

Predictor	VIF
Age	1.280
Sex	1.088
Chronic condition	1.048
Italian citizen	1.041
Mother relationship satisfaction	1.563
Father relationship satisfaction	1.778
Friendship satisfaction	1.163
Eating disorder risk	1.231
Prosocial score	1.235
Alcohol past 30 days	1.324
Nicotine past 30 days	1.399

Table S5. Comparison of OLS and robust MM regression estimates for SDQ TD outcome

	OLS						Robust MM					
Predictor	B_{OLS}	SE_{boot}	*CI* lower	*CI* upper	p_{BH}	β_{OLS}	B_{robust}	SE_{boot}	*CI* lower	*CI* upper	p_{BH}	β_{robust}
Age (yrs)	−0.39	0.34	−1.03	0.33	.362	−0.08	−0.67	0.40	−1.30	0.26	.122	−0.14
Sex (F)	2.30	0.70	0.91	3.70	.002	0.21	2.16	0.84	0.63	3.94	.081	0.20
Chronic condition	−0.16	0.68	−1.45	1.21	.788	−0.01	−0.49	0.89	−2.06	1.33	.528	−0.04
Italian citizen (yes)	0.43	1.00	−1.77	2.18	.788	0.02	0.26	1.08	−2.21	2.03	.824	0.01
Mother-rel. sat.	−0.68	0.56	−1.61	0.61	.362	−0.10	−0.88	0.80	−1.93	1.49	.309	−0.13
Father-rel. sat.	−1.00	0.47	−1.87	−0.03	.054	−0.18	−1.11	0.69	−2.19	0.16	.102	−0.19
Friendship sat.	−2.05	0.35	−2.71	−1.31	<.001	−0.33	−2.09	0.44	−2.83	−1.17	.031	−0.33
ED risk (yes)	1.39	0.87	−0.35	3.04	.210	0.11	1.45	1.04	−0.62	3.29	.186	0.11
Prosocial score	−0.64	0.20	−1.04	−0.27	.006	−0.23	−0.57	0.25	−1.17	−0.17	.102	−0.21
Alcohol 30 d (yes)	0.45	1.46	−2.24	3.56	.788	0.02	0.46	2.04	−3.13	4.89	.824	0.02
Nicotine 30 d (yes)	2.26	1.61	−0.77	5.55	.298	0.11	2.99	2.26	−1.40	7.22	.186	0.14

OLS: $F(11, 165) = 10.49, p < .001$

Note: Rel. sat. = relationship satisfaction; ED risk = eating-disorder risk.

Table S6. Comparison of OLS and robust MM regression estimates for SDQ TD15 outcome.

	OLS_{TD15}						Robust MM_{TD15}					
Predictor	B_{OLS}	SE_{boot}	*CI* lower	*CI* upper	p_{BH}	β_{OLS}	B_{robust}	SE_{boot}	*CI* lower	*CI* upper	p_{BH}	β_{robust}
Age (yrs)	−0.35	0.29	−0.93	0.23	.344	−0.08	−0.54	0.36	−1.14	0.35	.177	−0.13
Sex (F)	1.51	0.62	0.30	2.76	.034	0.16	1.47	0.74	0.07	2.99	.162	0.16
Chronic condition	−0.46	0.62	−1.64	0.79	.526	−0.04	−0.76	0.87	−2.29	1.03	.305	−0.07
Italian citizen (yes)	1.32	0.92	−0.60	2.97	.258	0.07	1.16	1.00	−0.94	2.93	.310	0.06

Mother-rel. sat.	−0.22	0.46	−0.97	0.87	.630	−0.04	−0.41	0.78	−1.24	2.99	.433	−0.07
Father-rel. sat.	−1.03	0.43	−1.84	−0.16	.031	−0.21	−1.10	0.73	−2.26	0.15	.145	−0.23
Friendship sat.	−1.27	0.33	−1.90	−0.62	<.001	−0.24	−1.23	0.41	−2.02	−0.47	.101	−0.23
ED risk (yes)	1.51	0.82	−0.10	3.13	.118	0.14	1.47	0.94	−0.40	3.29	.179	0.13
Prosocial score	−0.60	0.17	−0.95	−0.28	.004	−0.26	−0.56	0.23	−1.09	−0.19	.145	−0.24
Alcohol 30 d (yes)	0.63	1.30	−1.87	3.27	.630	0.04	0.92	1.81	−2.97	4.17	.516	0.06
Nicotine 30 d (yes)	1.89	1.42	−1.05	4.55	.297	0.11	2.58	1.96	−1.98	5.61	.200	0.14

OLS_{TD15}: $F(11, 165) = 8.38, p < .001$

Note: Rel. sat. = relationship satisfaction; ED risk = eating-disorder risk.

Table S7. Model fit statistics for OLS and robust MM regression models with SDQ TD and SDQ TD15 outcomes

Outcome	Model	*n*	*k*	R^2	Adj. R^2	RMSE	AIC	BIC	ψ	tuning	R^2_w	$n_e ff$	σ_{rob}
SDQ TD	OLS	177	11	0.412	0.372	4.20	1036.2	1077.5	—	—	—	—	—
SDQ TD	Robust MM	177	11	0.406	0.366	4.22	—	—	bisquare	4.685	0.487	161.3	4.090
SDQ TD15	OLS	177	11	0.359	0.316	3.72	993.1	1034.3	—	—	—	—	—
SDQ TD15	Robust MM	177	11	0.352	0.309	3.74	—	—	bisquare	4.685	0.431	160.9	3.590

Note. R^2 = coefficient of determination; Adj. R^2 = adjusted R^2; RMSE = root mean square error; AIC = Akaike Information Criterion; BIC = Bayesian Information Criterion; ψ = weight function used in the robust model; tuning = tuning parameter of the ψ function; R^2_w = weighted (robust) R^2; $n_e ff$ = effective number of observations after robust weighting; σ_{rob} = robust estimate of the residual standard deviation.

Fit statistics are reported for unstandardized models only. Standardized models were used exclusively for presentation of standardized coefficients (β) in Figure 1; their fit indices are identical to those of the unstandardized models. For robust MM regression, R^2 values are unweighted, and R^2_w reflects the weighted proportion of variance explained after down-weighting high-leverage and outlying cases.
